# The illusion of abundant communications and the ghost of *Red Lion*

**DOI:** 10.3389/frma.2022.1003481

**Published:** 2022-11-18

**Authors:** Michael J. Burstein

**Affiliations:** Benjamin N. Cardozo School of Law, Yeshiva University, New York, NY, United States

**Keywords:** net neutrality, communications law, common carriage, rate regulation, scarcity

## Abstract

Twentieth-century communications law was built on the assumption of scarcity-radio spectrum as a scarce natural resource and telephone networks as a natural monopoly. Scarcity justified both rate regulation and content regulation of the services offered over these communications resources. Telephone networks were subject to the nondiscrimination rules of common carriage, and the Supreme Court in *Red Lion Broadcasting v. FCC* famously upheld the “fairness doctrine,” which required that both sides of public issues be discussed fairly over broadcast media, expressly on the rationale that the scarcity of the airwaves justified content-based regulation under the First Amendment. As the century drew to a close, however, technological developments cast doubt on the assumption of scarcity and, therefore, much of the legal framework of communications law. In this chapter, I explain how both incumbent and startup providers reacted to this seeming technological abundance with acts aimed at creating or re-creating economic scarcity97strongly resisting encroachments on exclusive franchises or collusively slowing or halting the rollout of alternative networks97and how communications law has failed to keep up. It is widely acknowledged that our current statutory law is maladapted to modern technology, but in this work I recast the ongoing fights over net neutrality, affordable broadband, and platform speech regulation in terms of scarcity and abundance and argue that *Red Lion* is still with us in spirit97communications law should address the sources and effects of economic. I sketch out what such regulation might start to look like and conclude with some thoughts about what this story means for the central thesis of this volume.

Twentieth-century communications law was built on the assumption of scarcity. Radio spectrum was thought to be a scarce natural resource. Telephone networks were assumed to be natural monopolies. Scarcity justified both economic regulation and content regulation of the services offered over these media; if communications opportunities were scarce, it followed that they had to be regulated to ensure access. Telephone networks were therefore subject to the rate regulation and nondiscrimination rules of common carriage, and to a requirement of universal service. Broadcast media was licensed and the Supreme Court in *Red Lion Broadcasting v. FCC (1969)*^1^ famously upheld the “fairness doctrine,” which required that both sides of public issues be discussed fairly, on the ground that scarcity of the airwaves justified content-based regulation otherwise prohibited under the First Amendment.

As the century drew to a close, however, technological developments cast doubt on the assumption of scarcity and, therefore, much of the legal framework of communications law. The development of broadband infrastructure and the advent of packet-switched networks that enabled the delivery of multiple forms of content over multiple communications technologies gave rise to a widespread belief that bandwidth would no longer be scarce. Congress enacted the deregulatory Telecommunications Act of 1996^2^ in anticipation of such advances. But the promised abundance never came to pass. Instead, incumbent providers and startups alike reacted to technological abundance with acts aimed at creating (or re-creating) economic scarcity—strongly resisting encroachments on exclusive franchises, collusively slowing or halting the deployment of alternative networks, and engaging in other practices that make communications a luxury good. Even in the absence of technological scarcity, such practices can create scarcity-like conditions that lead to high prices and lack of access.

We are left with the worst of both worlds—communications law based on technological scarcity that no longer exists but poorly suited to the economic scarcity that incumbent providers have worked to create. It should be no wonder, then, that communications policy disputes have become some of our most intractable legal problems. Net neutrality—the principle that broadband Internet access service providers should not be permitted to change the terms of carriage for different users' content^3^—has been the subject of litigation for almost two decades as successive Federal Communications Commission decisions and court challenges go back and forth between interpretations of an old statue that poorly addresses a technology its drafters could never have anticipated. Meanwhile, the growth of large communications platforms with significant power over users' speech has scrambled traditional political positions, with some regulation-averse conservatives advocating for the imposition of common carriage-style nondiscrimination rules for those platforms,^4^ and Congress deadlocked over reforms to a key regulatory statute. All the while, rates for broadband Internet access service continue to increase and large swaths of the U.S. population remain without affordable broadband. The ghost of *Red Lion* is still with us—even in an era of bandwidth abundance, we fight over the terms of access to critical communications infrastructure.

This essay recasts the recent history of telecommunications regulation in terms of scarcity and abundance. As Desai and Lemley observe in the opening contribution to this volume, we ordinarily expect the reduction or elimination of scarcity to change the economics of production and distribution. “[S]pecial things happen,” they write, “when costs approach zero^5^.” To be sure, something special *has* happened—Internet applications and content have exploded into abundance. But the basic infrastructure of communications remains a choke point despite the technological promise of endless spectrum. Why? The answer lies in both a legal story and an economic story about the relationship between scarcity and abundance. The legal story is about what happens to laws designed for technological scarcity when that scarcity disappears. The economic story is about incumbents' reactions to the loss of that scarcity. Together, these stories tell us two things about the “abundance society” that is the subject of this volume. First, technological abundance does not necessarily equal economic abundance, and communications provides another case study—alongside copyright and other industries—of incumbents' attempts to replace technological scarcity with economic scarcity.^6^ Second, the law can and should respond to conditions of scarcity, whether they are technological or economic in nature. This last observation points to a way forward for communications law—to actively promote abundance.

## Communications law in the era of Scarcity

Communications in the 20th century was largely bifurcated into two technological mediums. One-to-one voice communications were carried over wired landline networks—the telephone system. One-to-many radio and television broadcasts were carried over radio spectrum. One important aspect of this architecture was the merger of content and infrastructure. Telephone service offered a singular means of communication over a single technology. Likewise, radio and television were offered only through the use of broadcast spectrum. Although different technological and economic conditions made the regulation of these two modes of communication somewhat different in turn, there were several common features to their 20th century regulatory paradigms.

What follows is a descriptive account of 20th century communications law that shows it to be broadly consistent with how one might regulate amidst conditions of significant technological scarcity. I do not claim that legislators and policymakers were expressly motivated by scarcity, though that was true in some cases.^7^ My more modest aim is to show that the landscape of 20th century regulation can be explained by reference to the scarcity of communications technology. Although they may not have been parsed in these terms at enactment, taken together these regulatory solutions represent a response to the technological scarcity of the underlying infrastructure.

Regulation of the use of radio spectrum was expressly driven by notions of scarcity and interference.^8^ The former referred to the fact that the radio spectrum had only a certain range of usable frequencies, and that certain frequencies were only well suited for certain uses—one could not use the same frequency for, say, television broadcasts and citizens band radio. The latter referred to the problem that multiple users attempting to use the same frequency in the same geography at the same time would interfere with one another and scramble each other's signals. As a result, two important entry restrictions were needed—allocation and assignment—and formed the basis of the 1927 Radio Act.^9^ Many of these restrictions survive today. “Allocation” means dividing the spectrum into usable frequency bands and allocating each band to a particular use. The FCC continues to employ a master “band plan,” under which the frequencies most suitable to a given use are reserved for that use and that use only.^10^ “Assignment” takes place within those bands, authorizing particular users to broadcast at particular frequencies within specified geographic areas.^11^ This prevents interference. Assignment is implemented through a licensing scheme in which the licensee has the exclusive right to the use of spectrum with certain physical and geographical characteristics.

Although there was historically significant debate over whether government should allocate and assign spectrum through an administrative process or a market-based process,^12^ there was little dispute that allocation and assignment were needed in some form. As Nuechterlein and Weiser write, “if the government just opened [the radio spectrum] up for a free-for-all tomorrow morning..., significant interference problems would likely impair people's ability to decode the signals sent by radio stations, cellular telephone providers, and ambulance dispatchers.”^13^

Scarcity also provided the particular constitutional basis for radio spectrum regulation. The airwaves were and are a critical forum for speech. In allocating and assigning spectrum, the government is effectively choosing who can speak through this medium. We ordinarily think the First Amendment does not allow the government to make such choices. The Supreme Court nevertheless upheld the government's authority to regulate access to spectrum in 1943, reasoning that “[u]nlike other modes of expression, radio inherently is not available to all. That is its unique characteristic, and that is why, unlike other modes of expression, it is subject to governmental regulation. Because it cannot be used by all, some who wish to use it must be denied.”^14^ Absent scarcity, the constitutional status of access regulation to wireless spectrum is in some doubt.^15^

Scarcity not only justified the regulation of access to the spectrum, but it also justified quite significant regulation of the content that was allowed over the public airwaves. The Communications Act requires the FCC to consider the “public convenience, interest, or necessity”^16^ Through the early 1980s, the Commission required radio and television broadcasters, as a condition of maintaining their licenses, to adhere to the “fairness doctrine”—“the requirement that discussion of public issues be presented on broadcast stations, and that each side of those issues must be given fair coverage.”^17^ This is a stark departure from First Amendment norms.^18^ It may be seen as a form of compelled speech. At the very least, it requires government to make choices about the kind of content broadcasters carry.^19^ It is likely not permissible in other contexts.^20^ But the Court in *Red Lion* held that spectrum is different.^21^ “Because of the scarcity of radio frequencies, the Government is permitted to put restraints on licensees in favor of others whose views should be expressed on this unique medium.”^22^ *Red Lion* therefore stands for the proposition that scarcity can justify significant encroachments on the ability of communications providers to choose which communications to broadcast.^23^

Telephone networks were regulated differently, and for different reasons, but the scheme of common carrier regulation that forms the core of the Communications Act can also be thought of as a response to a kind of techno-economic scarcity brought on by the confluence of technology, network effects, and the natural monopoly characteristics of telephone service. The copper wire-based transmission technology of the traditional telephone network was initially capable of carrying only analog voice traffic. Network effects meant that the value of the telephone network increased with each additional user. It is easy to see how this may be the case—“a lone telephone is of no practical value to its user because there is no one to call.... [W]ith each additional customer on the network[,] there are simply more people to call, and more people from whom to receive calls as well.”^24^ This implies that a network connecting everyone who wants to use the telephone is optimal. Now consider that historically the cost of building telephone networks was very high. “[T]o provide telephone service, a firm must incur a significant fixed investment... to build the initial network of switches, wires, and so on... but, once that investment has been made, the marginal cost of adding an additional phone customer is almost zero.”^25^ There were, moreover, no real technological substitutes for wireline telephony. Telephone service therefore tended toward natural monopoly in which the most efficient provision of services was by a single provider.

We could think of natural monopoly as a kind of techno-economic scarcity. If the economics of providing telephone service favor only a single network, then that is a serious restriction on both bandwidth and consumer choice. If there are no technological substitute, then even were additional providers to enter the market in an attempt to relieve that scarcity, they would face barriers to entry and high costs. In practice, telephone service was provided by the Bell system monopoly for much of the 20th century.

The regulatory response to the natural monopoly of telephone service was common carriage. Defining what is a “common carrier” is notoriously difficult.^26^ At common law, certain industries or services were granted monopoly status in return for accepting significant regulatory burdens, including limitations on charges, minimum service quality standards, and a requirement to accept all customers.^27^ Eventually, “these principles came to extend to any firm “affected with a public interest” that held itself open to the general public and purported to serve all comers.”^28^ Congress extended such regulation to telephone service in the Mann-Elkins Act of 1910^29^ and the Communications Act of 1934 established the basic outlines of common carrier regulation of the Bell system telephone monopoly.^30^ Telephone carriers must provide “communication service upon reasonable request,”^31^ and “[a]ll charges, practices, classifications, and regulations for an in connection with such communication service” must be “just and reasonable.”^32^ The FCC ensures that rates are just and reasonable by requiring common carriers to file tariffs that must be adhered to for all customers; private arrangements are not allowed.^33^ Finally, there is a strong nondiscrimination provision.^34^ Together, these provisions establish a system of pervasive rate regulation. The basic tradeoff the Bell system made was monopoly power in exchange for comprehensive regulation.

Because the Bell system was a regulated monopoly, an additional scarcity-related concern was that the monopoly would choose not to provide service in areas where it would be unprofitable to do so. “Universal service” was the solution to this problem. During the period of Bell System natural monopoly, the FCC and state regulators maintained a complex scheme of cross-subsidies and grant programs to ensure that the telephone network extended across most of the United States.^35^

Common carriage's requirement of nondiscrimination has long been thought to apply not only to the economic terms of an offer of telecommunications service, but also to the content that is carried over telephone wires. Telecommunications providers generally cannot discriminate on the basis of the content of users' speech.^36^ As Eugene Volokh writes, “Verizon can't cancel the Klan's recruiting phone number.... Certain kinds of important infrastructure under [the rules of common carriage] are available equally to all speakers.”^37^ This again can be seen to arise from scarcity. If a national platform for communications is scarce, then it is reasonable to think that the government should require it to be open to all.^38^

Although the telephone system and broadcast media were technologically quite distinct, they were regulated in similar ways. Both technologies could be seen as scarce resources—the airwaves due to limited spectrum and interference, and the phone wires due to the natural monopoly characteristics of the service and the absence of technological substitutes. Both technologies were subject to economic regulation that can be seen as a response to scarcity—the system of radio spectrum licensing, and common carriage rate regulation coupled with a universal service obligation. And both technologies also were subject to content regulation that can be seen as a response to scarcity—the fairness doctrine, and common carriage nondiscrimination. This paradigm stood for the better part of the 20th century.^39^

## Deconstructing scarcity

In the late 1990s and early 2000s, it became common to speak of the end of scarcity in communications. Technological developments suggested that the bottlenecks posed by copper plant technology and natural monopoly in wireline services and the problems of scarcity and interference in the radio spectrum would no longer define the market for communication services. This was always somewhat more theory than fact—it was then,^40^ and it remains so now. But to the extent that technological conditions were and are in place to reduce or eliminate scarcity, the choice to implement laws and policies that encourage abundance to bloom is distinct.^41^ Communications law has not adapted to the evanescence of technological scarcity. The Telecommunications Act of 1996 was intended as a transitional regime to encourage competition in local telephone markets,^42^ but largely failed to anticipate the growth of the Internet or the communications technologies that support it. It is built on roughly the same regulatory foundations that prevailed during the period of technological scarcity. To the extent it addressed the Internet and related technologies at all, it envisioned the Internet existing in a world where abundant communications was not merely theory but also reality, a world in which regulation was largely unnecessary.

### The theory of abundant communications

The theory of abundant communications has two components to it. The first is technological developments in the underlying communications infrastructure that have created significantly more bandwidth—the ability to carry more information through wires or over the air. The second is innovation in the way that information itself is transmitted and the development of packet-switched rather than circuit-switched networks. These two developments together have created the technological conditions for the end of communications scarcity.

#### The bandwidth explosion

The technology underlying wireless transmission of data was completely transformed throughout the decade of the 1990s. Prior to that time, the primary consumer use for wireless spectrum was one-way communication: radio and television broadcasters would transmit audio or video signals over the airwaves that consumers would receive on their home equipment. Some specialized applications were enabled for two-way communication, like citizens band radio and the radio systems used by government and first responders.

The widespread adoption of two-way over-the-air communication required the development of cellular technology.^43^ This technology “uses spectrum more efficiently by enabling networks to reuse the same frequencies” by managing the flow of data traffic among different “cells” throughout the network.^44^ This network management technique created the possibility for much faster and more accurate transfer of information. In the decades since the first cellular networks arose in the 1980s, successive waves of technological development have improved both speed and efficiently. Modern cellular networks (“LTE” or “long-term evolution” networks) use a variety of protocols to increase the efficiency of data transmission within a given range of frequencies.^45^ As technology has improved, the FCC has made more spectrum available for these more efficient uses.^46^

Cellular technologies are not the only developments in wireless communications that have ushered in an era of skepticism about natural limits to communications. Wi-Fi, a popular protocol for transmission over local networks, relies on the use of unlicensed spectrum and a management method known as “spread spectrum.” The ability to implement spread spectrum over unlicensed spectrum has fundamentally changed the economics of wireless technology.^47^ It has meant near-ubiquitous access to high speed networks capable of transmitting all manner of data, as described below.

Similar improvements have taken place with respect to wireline infrastructure—both the infrastructure that interconnects networks themselves and that which enables transmission down the “last mile” to consumers. The traditional telephone network last-mile was made up primarily of copper wire, which had limited capacity. It could transmit voice reasonably well at reasonably low cost—hence its use for telephone service. But it needed special equipment known as DSL to enable it to carry data at high speeds. The architecture of cable networks, whose last-mile connections were built primarily from coaxial cable, enabled higher bandwidth suitable to multichannel video offerings. Cable networks proved to be more suitable than telephone networks to the transmission of data in all of its forms and now are the basis for most consumers' retail connections to the Internet. But the possibility of even higher bandwidth retail connections—fiber optic cable running to the home—made it realistic to think that widespread consumer adoption of super-high bandwidth connections was imminent.

This optimism was aided by a concurrent decline in construction costs for telecommunications networks that suggested the natural monopoly conditions were easing. A group of well-funded companies called cable “overbuilders” launched ambitious plans to build second cable networks in many municipalities that had previously been served only with an exclusive franchisee drawn from the legacy cable companies.

Finally, the 2000s saw the development of a number of competing technologies for the transmission of broadband data. Broadband over powerline (BPL) technology would utilize the existing electrical wiring in residences and offices to enable data transmission using conventional outlets. Satellite broadband could provide an alternative to terrestrial solutions just as it did for the transmission of television signals. And various forms of fixed wireless communication that were different from the cellular model were piloted and showed promise.

While not all of these technologies succeeded, nor did the successes necessarily develop as expected,^48^ it is fair to say that the technology exists to provide near-universal access to high speed data transmission. In other words, we have reached the point where technology exists to render limitations on bandwidth largely irrelevant to the provision of communications services.

#### Decoupling applications and content from transmission—the rise of the “layers” theory of communications

The second major technological development was the ability to transmit all forms of communication as data. This is often described as a change from circuit-switched to packet-switched networks.^49^ In the former arrangement, which typified 20th-century telephone service, there is a direct transmission path established between the two parties that want to exchange data. By contrast, the data in packet-switched networks is broken into small “packets” with an instruction about where they should be routed and how they should be reassembled. The individual packets are then each sent along the most efficient route, as determined dynamically, from one end-user to another. The technical details are less important for thinking about scarcity and abundance in telecom than the core concept: any type of data can be made into packets and sent and received through a packet-switched network. This includes voice, video, and data. The core functionality of the Internet is the use of protocols for routing this data.^50^

This development fundamentally changes the way we think about communications. It decouples the telecommunications service from the underlying infrastructure. Telephone service—or, at least, person-to-person voice communication—does not have to be provided over telephone lines by telephone companies. Radio and television service is no longer restricted to over-the-air broadcasts. And, of course, the ability to transmit data in any form has resulted in a wide range of Internet-based applications that would never have been conceived in the world of traditional communications infrastructure. Once data is packetized, it becomes relatively indifferent to the physical medium over which it is transmitted.^51^

The more sophisticated way to conceive of modern communication is as a series of layers.^52^ The details of various layered models of the Internet and other forms of communication are intricate and continue to be the subject of debate. But the most commonly-invoked “simplified model [has] four distinct layers, visualized vertically and adjacently in a “stack” format.”^53^ At the bottom of the stack is the *physical* layer—the physical infrastructure that makes up modern communications networks. This includes all of the equipment necessary for wired and wireless communications from the home to the network. Next is the *network or protocol* layer, which comprises the various protocols that tell the packets described above where to go. Then comes the *application* layer which “facilitate[s] the delivery of content to and from users.”^54^ Email, streaming video, instant messaging, voice-over-IP, videoconferences all are various applications offered by a multitude of service providers. At the top of the stack is the content layer—the individual pieces of content such as the individual messages sent through email or individual videos offered by a streaming video provider.^55^

The theory of abundant communications turns in large part on the separation of the physical and application layers. If applications (and, in turn, the content they deliver) can run on any physical network, then the ability to reach end users depends solely on the availability of bandwidth in the physical layer. If technology exists to render that availability infinite, then whether access to the physical layer remains a bottleneck depends on economic and policy choices. That is the subject to which I turn next.

### Communications law in the theory of abundance

Communications law likely looks quite different if it is drawn to abundance rather than scarcity. As described above, the 20th-century paradigm can be explained in terms of technological scarcity. Without that scarcity, the underpinnings of many traditional communications regulations are called into question. Take common carrier rate regulation, for example. If the physical layer is no longer a natural monopoly, then there is not necessarily a reason for rate setting. A competitive market for transmission services would set prices appropriately and applications would flourish on those services. Spectrum allocation may continue to be necessary, but within bands dedicated for the provision of retail communications services, assignment to particular licensees may disappear as technological solutions to interference dominate regulatory solutions. Universal service obligations could be met not by requiring it of providers but rather by subsidizing needy consumers who would have a number of choices.

So too with respect to nondiscrimination rules. The abundance of bandwidth could mean the proliferation of networks with a variety of architectures. Some may be relatively closed—networks optimized for a particular purpose that are free to discriminate against applications or content that may detract from that purpose. Others may be relatively open, like the basic Internet, on which applications may operate freely under a norm of non-discrimination, even if it is not imposed as a regulatory matter. Christopher Yoo argues that consumer welfare may in fact be enhanced by allowing innovation *in network design* to flourish in the absence of a non-discrimination rule.^56^ Corollary to this argument is the idea that if sufficient consumer demand exists for a non-discrimination norm, then in an era of abundant communications such a network will emerge alongside networks that follow other rules.^57^

The Telecommunications Act of 1996^58^—the most significant revision to communications law since 1934—did not quite enact this deregulatory scheme. At least, it did not do so deliberately. But intentionally or not, much of what the FCC now calls “broadband Internet access service,”—“a mass-market retail service by wire or radio that provides the capability to transmit data to and receive data from all or substantially all Internet endpoints”^59^ is regulated with the minimal intervention described above.

The 1996 Act was enacted against a backdrop of evidence that telephone service was becoming competitive. The growth of a competitive market for long-distance service and the rise of cellular communications showed that natural monopoly was not an inevitable market structure.^60^ Much of the statute was aimed at “roll[ing] back” the assumption of natural monopoly in local phone markets and encouraging a transition to competitive provision of local telephone service.^61^ It also aimed to reduce the regulatory barriers to competition, such as the structural separation of local from long distance service.^62^ Finally, it sought to formally implement a competitively neutral universal service plan.^63^

Importantly, the 1996 Act made these changes within the existing framework of service-based regulation. The transition to competition in telephone service assumed that telephone service would continue to exist as a stand-alone offering. So too did the 1996 Act preserve regulatory distinctions between telephone, radio (including mobile telephony), and cable. The Internet was in its infancy. Broadband Internet service barely existed. The decoupling of applications from infrastructure had largely not yet occurred. As Nuechterlein & Weiser explain, “Congress did not foresee that cable and telephone companies would compete” in the market for broadband Internet service, so “it did not set forth a clear regulatory framework for that market.”^64^ Indeed, to the extent the 1996 Act mentions the Internet at all, it is primarily in policy statements that generally do not carry with them the direct authority to regulate,^65^ but that nevertheless evince a distinctly deregulatory stance.^66^

Broadband Internet access service therefore occupies a statutory netherworld. As described above, the Communications Act divides regulatory approaches by service. “Telecommunications service” is “the offering of telecommunications for a fee directly to the public,”^67^ where telecommunications is defined as “the transmission, between or among points specified by the user, of information of the user's choosing, without change in the form or content of the information as sent or received.”^68^ Telecommunications service is regulated under Title II of the Act, which gives the FCC the full range of common carrier authorities described above; it is the part of the Act that governs the traditional telephone network. By contrast, “information service,” defined as, “the offering of a capability for generating, acquiring, storing, transforming, processing, retrieving, utilizing, or making available information *via* telecommunications,”^69^ is not pervasively regulated. It falls only under the FCC's general authority in Title I of the Act to “perform any and all acts, make such rules and regulations, and issue such orders, not inconsistent with this chapter, as may be necessary in the execution of its functions.” That authority only “enables the Commission to regulate on matters “reasonably ancillary to the... effective performance of its statutorily mandated responsibilities.”^70^

The FCC's ancillary authority is highly limited in scope.^71^ Information services regulated under Title I of the Act are therefore subject only to light regulation.^72^ The FCC lacks the authority to regulate information services in the comprehensive manner by which it regulates traditional telephone, radio, and cable services. In the absence of such direct authorities, the Title I regulatory regime looks much like the theoretical law of abundant communications sketched out above. It lacks access regulation and a nondiscrimination rule.^73^ As described in more detail below, the central question of broadband policy is whether it is properly classified as a telecommunications service subject to heavy regulation or an information service subject to light regulation.^74^ Suffice for now to say that current law creates the possibility that broadband would be unregulated as if the restraints of scarcity were lifted and, at the very least, creates uncertainty about the scope of broadband regulation under any rationale.

## Reconstructing scarcity

Although the technological conditions may exist for abundant communications, market and political barriers have kept most consumers from realizing its benefits. Perhaps unsurprisingly, the reaction of incumbent communications providers to the end of technological scarcity has not been to embrace competition and extend the fruits of abundance to all Americans. Instead, incumbents have largely used their market positions to erect economic barriers to abundant communications even in the absence of technological barriers. In so doing, they have ushered in an era of renewed scarcity, this time economic rather than technological.

Start with wired communications. In the mid- to late-2000s, it became clear that DSL technology had reached its limit for broadband speeds, whereas cable technology had not. If the incumbent telephone providers were to compete with the cable companies for the retail broadband market, they would have to deploy new networks. The two largest incumbent wired telcos—Verizon and AT&T—both announced plans to build fiber optic networks that would deliver much faster Internet and enable voice, video, and data content to be transmitted over a single platform. AT&T's “U-verse” product would use a “fiber to the curb” model, where the high-bandwidth fiber ran to the customer premises, but then traditional coaxial cable would run into the house. Verizon's “Fios” product was a “fiber to the home” model in which fiber was used for the entirety of the last mile. Both companies began deployments but never completed their ambitious build plans. Verizon, for example, promised New York City that it would build fiber connections to all of the 3.1 million households it served with traditional telephone service, but halted construction after passing only 2.2 million households.^75^ Nationwide, Verizon announced in 2010 that it was completing planned builds and would continue to service existing customers but would not engage in significant expansion.^76^

The telcos blamed high capital costs for their decision to terminate the buildout of new fiber optic networks. But both Verizon and AT&T found that their mobile businesses were more consistent sources of growth and profitability than their declining landline businesses. While the telcos were beginning to compete with cable providers in broadband Internet access, the cable providers threatened to enter the lucrative mobile phone market. In an arrangement that some have labeled “collusion,”^77^ Verizon and four major cable providers agreed to cross-market each other's services in areas where they did not directly compete. This arrangement effectively removed incentives to continue building the fiber network.

The other significant threat to cable broadband came from competitors using similar technology but taking advantage of lower construction costs. The cable “overbuilders” were a group of companies that sought additional cable franchises in municipalities where incumbent cable companies already provided service. The incumbents lobbied furiously against such franchises. They also lobbied against government provision of fiber networks or other public-focused overbuilds.^78^

The result is that the market for wireline broadband is not competitive. At the highest commercially available broadband speeds, more than 50% of the country has access only to one provider.^79^ At mid-tier speeds, more than 75% of the country has access to two or fewer fixed broadband providers.^80^ The majority of those providers are incumbent cable companies operating pursuant to exclusive local franchises.^81^ Fiber makes up only 16% of residential broadband connections, less even than the number of old DSL connections.^82^ The US has historically lagged, and continues to lag, other countries in fiber deployment.^83^ The U.S. currently ranks 32nd amongst the 39 OECD countries for the percentage of fiber-based home connections.^84^ Although technological abundance is possible through fiber-to-the-home, the economic structure of the wireline broadband industry has tended once again toward monopoly, relying primarily on incumbent cable infrastructure. As a result, U.S. broadband connections remain generally slower^85^ and more expensive^86^ than other comparable nations.

Mobile broadband has of course exploded in usage and popularity.^87^ But consolidation in the mobile broadband industry has been significant. There are now only three major providers of cellular phone service: Verizon, AT&T, and T-Mobile. They have a 99% market share.^88^ This raises at least two problems. First, mobile broadband pricing in the United States remains high, which poses a significant access challenge for under-served communities. Second, it is unclear whether or when mobile broadband will truly be a substitute for fixed broadband speed and capabilities. The rollout of enhanced speeds from first 4G LTE and now 5G networks has been slower in the United States than elsewhere. To give just one example, a condition of the recently approved merger between Sprint and T-Mobile was that the combined entity must reach 97% of the U.S. population with 5G networks within 6 years.^89^ By contrast, that coverage level is predicted to take much less time in other countries.^90^ Even where 5G networks are available in the U.S., they appear to be significantly slower than their peers elsewhere.^91^ Broadband speed is a key determinant of its utility for streaming video and other applications; until the widespread deployment of 5G networks, it is difficult for mobile to compete with fixed broadband. But the industry consolidation described above may pose a barrier to the rapid implementation and diffusion of 5G technology.

Meanwhile, other technologies that showed promise have achieved little adoption. Satellite broadband and fixed wireless make up only small percentage of broadband connections,^92^ and broadband over powerline never succeeded.

## Toward a communications law for the era of renewed scarcity

In 2022, we are left with a regulatory quandary. The decoupling of the application and transmission layers has led to abundance in the former, but a significant bottleneck in the latter. Internet-enabled applications have proliferated and have an enormous influence on our daily lives. They are also a domain of significant innovation. By contrast, the market for transmission services is an oligopoly, with insufficient competition, a slow pace of innovation, and high prices. This poses two problems. The first is that the public is deprived of the benefits of access to ubiquitous and affordable high speed internet service. The second is that providers in the physical layer may leverage their market power to stifle innovation in the applications layer. In other words, the physical layer has become a significant bottleneck.^93^

The current statute is insufficient to solve these problems. It was written for an age of technological scarcity. It is maladapted to an era in which scarcity is the result of market actors' economic choices. Take as an example one of the central problems in contemporary telecom policy: net neutrality. Network neutrality generally refers to “the principle that broadband providers must treat all internet traffic the same regardless of source.”^94^ Although the specifics of net neutrality policies may differ,^95^ most include some combination of what the FCC implemented in its *2015 Open Internet Order*: rules prohibiting broadband providers from “blocking lawful content, applications, services, or non-harmful devices,”^96^ from “impairing or degrading lawful Internet traffic on the basis of content, application, service, or use of non-harmful device,”^97^ and from engaging in “paid prioritization,”^98^ or contracts to prioritize traffic from certain sources over that from others. Together, these rules keep the Internet open. They make it so that any applications or content providers can have reasonable access to the physical layer infrastructure necessary to reach wide audiences. They do not have to bargain with monopolist broadband providers for access or preferred access. They do not have to compete on an uneven playing field with applications or content affiliated with monopolist broadband providers. This is often thought to be the cornerstone of innovation on the Internet.^99^

The (very, very) long history of net neutrality regulation and litigation^100^ reveals two problems with the current statute. First, as described above, broadband Internet access service has alternately been classified as a “telecommunication service” subject to the full range of common carrier regulations in Title II of the Act and an “information service” subject to Title I of the Act. Because the Supreme Court has held that this classification decision is for the FCC to make,^101^ different FCCs in different presidential administrations have come to different conclusions based on their policy preferences. The result is significant instability. To wit, broadband Internet access service was classified as an information service from the Bush administration in 2002^102^ until the Obama administration reclassified it as a telecommunications service in 2015.^103^ The Trump administration changed course again in 2018, reclassifying broadband as an information service.^104^ Net neutrality is now in a state of limbo, with some states moving to enact rules of their own in the absence of clear federal authority.^105^

Second, as a matter of substance and assuming that one supports net neutrality, neither Title I nor Title II provides a wholly sound basis for implementing the policy. The D.C. Circuit has held that while Title I ancillary authority enables the FCC to promulgate *some* kind of open Internet rule, it does not provide the authority to implement the no-blocking, no-throttling, and no-paid-prioritization rules that form the heart of net neutrality, as described above.^106^ Title II, on the other hand, is over-inclusive. Although it grants the FCC the authority necessary to enact net neutrality rules—really, a species of common carrier nondiscrimination rules—it also authorizes the FCC to engage in the same kind of deep economic regulation that it applied to telephones. In the *2015 Open Internet Order*, the FCC invoked its “forbearance” power to decline to enforce full common carrier tariff-based rate regulation on broadband service providers.^107^ Common carrier regulation is generally thought to be inappropriate if applied in full to broadband internet access service. Although there is a case for some rate regulation of broadband service, particularly given the economic scarcity described above, the contours of such regulation are sufficiently different from traditional tariff-based telephone or cable rate regulation that new statutory authority would likely be necessary.^108^

Given the retreat (even if not complete) of technological scarcity but the persistence of economic scarcity—barriers to access to the best communications infrastructure—new legal approaches to regulation should aim squarely at the latter. The motivating goal should be to ensure that the fruits of technological abundance have as few economic barriers to consumption as possible. In other words, communications law should promote abundance.^109^ It can, in theory, do this through two broad mechanisms: conduct rules to reduce market barriers to abundance, and spending to affirmatively promote abundance. Although sketching out a complete Telecommunications Act of 2022 is well beyond the scope of this essay, a few examples can demonstrate the point. On the regulation side of the ledger, rate regulation and net neutrality help remove incumbents' ability to put up barriers to abundance. They do so in ways that are reminiscent of common carriage, but technologically neutral—promoting economic access and ensuring nondiscrimination. Several states have passed laws prohibiting government-funded provision of broadband services or otherwise restricting competition.^110^ Federal preemption of such laws would remove another barrier to abundance. On the spending side, the government has many tools at its disposal to promote abundant communications. The recently enacted Infrastructure Investment and Jobs Act^111^ allocates $65 billion of investment in broadband for a variety of purposes, but most notably for the buildout of broadband infrastructure where it currently does not exist or under-serves particular communities and for subsidies to consumers to defray the cost of broadband service.

The precise mix of abundance-promoting policies, of course, requires deeper study of the particular circumstances that give rise to economic scarcity. But the policies described above represent steps toward resolving the most significant sources of economic scarcity described in Part III—consolidation and lack of choice. In an oligopolistic or monopolistic environment, net neutrality prevents significant departure from the nondiscrimination norm central to access to communications infrastructure; appropriately tailored rate regulation facilitates consumer access to broadband across income levels; and spending on broadband infrastructure facilitates access across geographies. Together these policies help dismantle economic barriers to technological abundance.

## Concluding thoughts: Scarcity, regulation, and abundant communication

The recent history of the communications industry teaches some important lessons about the relationship between scarcity, abundance, and regulation. First, technological abundance does not necessarily equal economic abundance. It is still largely correct that we live in a world with abundant bandwidth. But the market structure of the telecommunications industry has maintained economic scarcity. Second, even when technological scarcity begins to abate, economic scarcity can be *created* by incumbents. The story of communications I tell in this essay is one of *reaction* to technological change. The players in the industry acted to stifle abundance and promote scarcity. That leads to the third lesson, that policy can affirmatively encourage abundance and reduce scarcity if we choose to do so. The ghost of *Red Lion* still haunts the modern communications landscape. Although the technological scarcity rationale on which it was based has largely disappeared, it remains important to recognize the sources of scarcity in the communications environment and take steps to promote abundance.

These lessons are consistent with the observations made in several other contributions to this volume. That technological scarcity may be replaced with economic scarcity is a theme that can be explored in copyright law, with respect to NFTs, and in a host of regulated industries. The relevant questions to ask are what *kind* of scarcity, if any, is created in modern technology-enabled industries, and what policies might promote abundance instead? Answering these questions should lead us not to replicate the responses to scarcity of the past but rather to embrace the possibility of abundance in the future.

## Data availability statement

The original contributions presented in the study are included in the article/supplementary material, further inquiries can be directed to the corresponding author/s.

## Author contributions

The author confirms being the sole contributor of this work and has approved it for publication.

## Conflict of interest

The author declares that the research was conducted in the absence of any commercial or financial relationships that could be construed as a potential conflict of interest.

## Publisher's note

All claims expressed in this article are solely those of the authors and do not necessarily represent those of their affiliated organizations, or those of the publisher, the editors and the reviewers. Any product that may be evaluated in this article, or claim that may be made by its manufacturer, is not guaranteed or endorsed by the publisher.

## Footnotes

^1^Red Lion Broadcasting v. FCC [hereinafter “Red Lion”].

^2^Pub. L. No. 104–104, 110 Stat. 56 (1996) (“1996 Act”).

^3^The term “net neutrality” is broadly credited to Tim Wu. See Wu (2003). For a comprehensive treatment of the principle, see van Schewick (2010).

^4^*See, e.g.*, Biden v. Knight First Amendment Inst. and at Columbia Univ; Volokh (2021).

^5^Desai and Lemley (2022).

^6^It is worth pausing for a moment to clarify my use of these terms. I use “technological scarcity” to mean limitations on the ability to communicate given the immutable characteristics of available technology at the time. I treat this variable as largely exogenous. “Economic scarcity,” by contrast I treat as endogenous. It arises from market choices that raise prices and reduce access. While technological scarcity is not immune to policy responses, I posit that economic scarcity is more easily remedied and its persistence is therefore more commonly a *choice*; in most cases, it represents a policy failure.

^7^For discussion in the context of radio spectrum regulation, see generally Hazlett (1990).

^8^*See* Benjamin et al. (2001).

^9^*See id*. at 11–23.

^10^*See* Nuechterlein and Weiser (2007).

^11^*See id*. at 235–239.

^12^*See, e.g*., Coase (1959) (arguing that market methods would yield more optimal use of spectrum than government decision making).

^13^Nuechterlein and Weiser, *supra* note 10, at 230.

^14^National Broad. Co. v. United States (1943).

^15^See, e.g., Spitzer (1989) and Benkler and Lessig (1998).

^16^47 U.S.C. § 307(a).

^17^Red Lion at 369.

^18^Similarly, the statute requires—still—that candidates for public office be granted equal opportunities to access broadcast media. *See* 47 U.S.C. § 315.

^19^*Cf*. Desai (2015) (noting that it is “an open question... exactly who should decide to what someone should be exposed,” and that requiring “editors, station programmers, bloggers, online news, search services, social networks and more must take space to offer low-interest stories of opposite views of the core audience is still to require *someone* to decide what those other offerings should be”) (emphasis added); Hazlett et al. (2010) (describing both political parties' use of the fairness doctrine).

^20^*See, e.g*., Miami Herald Pub. Co. v. Tornillo (newspapers) and Turner Broad. Sys (1994) (cable).

^21^*See* Red Lion at 386 (“Although broadcasting is clearly a medium affected by a First Amendment interest, differences in the characteristics of new media justify differences in the First Amendment standards applied to them.”) (citation omitted).

^22^*Id*. at 390.

^23^The FCC repealed the fairness doctrine in the mid-1980s. *See* In the Matter of Inquiry into Section 73.1910 of the Commission's Rules and Regulations Concerning the General Fairness Doctrine Obligations of Broadcast Licensees (1985); In re Complaint of Syracuse Peace Council 2 FCC Rcd 5043 (1987). Although *Red Lion* remains technically good law, its premises have largely been undermined as a factual matter, *see infra* notes 45–52 and accompanying text, and its continued viability has been questioned as a legal matter. *See* FCC v. Fox Television Stations and 556 (2009). For scholarly criticism of Red Lion, *see* Yoo (2003).

^24^Benjamin et al., *supra* note 8, at 615–616.

^25^*Id*. at 617.

^26^*See* Yoo (2021) and Biden v. Knight First Amendment Inst. and at Columbia Univ.

^27^*See* Huber et al. (1999); Yoo, *supra* note 23, at 466–475 (identifying “considerations that have historically been used to define common carriers” to include “market power, whether an industry is ‘affected with the public interest,' whether the entity regulated is part of the transportation or communications industry, whether it receives countervailing benefits from the government, and whether the actor holds itself out as providing service to all”).

^28^Huber et al., *supra* note 27, at 13–14.

^29^*See id*. at 16.

^30^*See id*. § 3.11.

^31^47 U.S.C. § 201(a).

^32^47 U.S.C. § 201(b).

^33^47 U.S.C. § 203.

^34^47 U.S.C. § 202(a).

^35^*See* Huber et al., *supra* note 27, §§ 6.1–6.2.

^36^*See id*. § 14.6.6; Biden v. Knight First Amendment Inst. and at Columbia Univ.

^37^Volokh (2021); *see also id*. at 384–385.

^38^There is some history of statutes and cases allowing phone companies to discriminate against dial-a-porn services, but those cases involved statutory derogations from the common law of common carriage. *See id*. at 384 n.20.

^39^To be sure, new technologies arose and were accounted for in the skein of communications law. Cable is the most significant example, warranting eventually its own section of the Act (what is now known as Title VI). Cable regulation has a long and complicated history, beginning with the FCC's efforts to regulate community access television antennas through its ancillary jurisdiction, and eventually leading to the enactment of a sui generis federal statutory regime that incorporates some elements of Title II but relies more significantly on cooperative federalism through local regulation of cable franchises.

^40^*See, e.g*., Hazlett (2001).

^41^*See* Desai and Lemley, *supra* note 5, at 34–36 (drawing distinction between scarcity-reducing technology in the energy sector and market/political hurdles to implementing it).

^42^*See* Nuechterlein and Weiser*, supra* note 10, at 69–74.

^43^*See* Dodd (2019).

^44^*Id*.

^45^*See id*. at Tbl 7.2 for a description of the modern variations of LTE networks.

^46^*See id*. at 327.

^47^*See* Benkler (2002).

^48^*See infra* Part III.

^49^*See* Dodd, *supra* note 43, at 23–25; Nuechterlein and Weiser, *supra* note 10, at 40–45.

^50^*See id*.

^51^I say “relatively” here because it still remains the case that different types of data are subject to different network performance tolerances. Video, for instance, is far more data intensive than text and therefore requires more bandwidth. In order for the experience of watching a video to remain tolerable, moreover, there must be little interruption and little “latency,” or network delays. This is even more true of simultaneous videoconferencing. Because of these different tolerances, there is an argument that networks can be differentiated by their performance standards and optimized for particular technologies. *See, e.g*., Wu and Yoo (2007).

^52^*See, e.g*., Benkler (2000), Lemley and Lessig (2001), Werbach (2002), and Solum and Chung (2004).

^53^Reid (2020).

^54^*Id*.

^55^*See id*. at 612–613.

^56^*See* Yoo (2009).

^57^*See id*. at 212–215.

^58^Pub. L. No. 104–104, 110 Stat. 56 (1996).

^59^In the Matter of Protecting and Promoting the Open Internet (“^2015^, Open Internet Order”).

^60^*See* Nuechterlein and Weiser, *supra* note 10, at 69–70.

^61^It did so by requiring incumbent local exchange providers—the legacy Bell companies—to allow interconnection by competing networks, *see* 47 U.S.C. §§ 251–254, and to allow competitors to lease “unbundled” elements of their local networks, *see id*. § 252. These provisions were the subject of extensive litigation between incumbents and competitors.

^62^*See id*. § 253.

^63^*See id*. § 254.

^64^Nuechterlein and Weiser, *supra* note 10, at 73.

^65^The most significant section that concerns the Internet is § 706 of the 1996 Act. That section provides that the FCC “shall encourage the deployment on a reasonable and timely basis of advanced telecommunications capability to all Americans... by utilizing, in a manner consistent with the public interest, convenience, and necessity, price cap regulation, regulatory forbearance, measures that promote competition in the local telecommunications market, or other regulating methods that remove barriers to infrastructure investment,” 47 U.S.C. § 1302(a), and requires the FCC to conduct an annual survey of broadband deployment, *id*. § 1302(b). In Verizon v. FCC, the D.C. Circuit held that § 706 of the 1996 Act constitutes a grant of regulatory authority. *See id*. at 636–642. But as described above, that grant of authority is highly circumscribed. *See id*. at 649–651 (holding that the FCC cannot impose common carrier-like obligations on broadband Internet access service providers).

^66^*See, e.g*., 7 U.S.C. § 230(b)(2) (“It is the policy of the United States... to preserve the vibrant and competitive free market that presently exists for the Internet at other interactive computer services, unfettered by Federal or State regulation.”).

^67^47 U.S.C. § 153(53).

^68^*Id*. § 153(50).

^69^*Id*. § 153(24).

^70^Mozilla Corp. v. FCC (quoting American Library Assn. v. FCC).

^71^*See, e.g*., Comcast Corp. v. FCC (holding that ancillary authority could not support imposition of “net neutrality” on broadband information service providers).

^72^This is consistent with the FCC's treatment of “enhanced services,” the regulatory forerunner to the statutory “information services” category. As communications providers gradually began incorporating early computer technology into communications networks, the FCC was wary of cutting off technological innovation. *See* United States Telecom Association v. FCC, for a succinct description of this historical approach.

^73^*See* Verizon v. FCC at 636–642.

^74^Though I have argued elsewhere, including in litigation over the FCC's net neutrality rules, that broadband Internet access service is a telecommunications service. *See* Brief of First Amendment Scholars as *Amici Curiae* in Support of Respondents, United States Telecom Assn. v. FCC (2016).

^75^*See* Brodkin (2020). New York City sued Verizon to enforce the terms of its franchise agreement and the parties settled out of court.

^76^*See* Svensson (2010).

^77^*See, e.g*., Dampier (2012).

^78^*See* Sallet (2019).

^79^*See* In the Matter of Communications Marketplace Report (“^2020^, Communications Marketplace Report”).

^80^*Id*.

^81^*See id*. ¶ 86 fig. II.B.1.

^82^*See id*.

^83^*See generally* Berkman Center for Internet Society (2010).

^84^OECD Broadband Portal §, 1.10 (2021).

^85^*See id*. § 2.1.

^86^*See id*. § 4.10; Berkman Center for Internet and Society, *supra* note 83, at 182-200.

^87^See Desai (2014) and sources cited therein.

^88^*See* 2020 Communications Marketplace Report, *supra* note 79, at ¶ 22 fig. II.A.3.

^89^*Id*. ¶ 81.

^90^*See* Sag (2022).

^91^*See* Fletcher (2021).

^92^*See* 2020 Communications Marketplace Report ¶ 125.

^93^*See* Nuechterlein and Weiser*, supra* note 10, at 151–158, for a discussion of the history of attempts to prevent monopoly leveraging in the interaction between communications and computing.

^94^United States Telecom Association v. FCC.

^95^*See* van Schewick, *supra* note 3, at 220–221 (describing variations of net neutrality proposals).

^96^*2015 Open Internet Order* ¶ 105 (“No-Blocking”).

^97^*Id*. ¶ 106 (“No-throttling”).

^98^*Id*. ¶ 107.

^99^*See* van Schewick, *supra* note 3, for a comprehensive discussion.

^100^For a capsule summary of this history, first read United States Telecom Association v. FCC, and then read Mozilla Corp. v. FCC.

^101^*See* National Cable and Telecomms. Assn. v. Brand X Internet Servs and Mozilla Corp. v. FCC at 19–22; United States Telecom Association v. FCC at 701–705.

^102^There is some complexity here too. The FCC originally classified DSL as a telecommunications service, in 1998, and cable modem service as an information service, in 2002. Following National Cable and Telecomms. Assn. v. Brand X Internet Servs (2005), the Commission reclassified all broadband services as information services. *See* United States Telecom Association v. FCC at 691–693.

^103^*See* 2015 Open Internet Order.

^104^In re Restoring Internet Freedom (2018); *see* Mozilla Corp. v. FCC at 17.

^105^California enacted its own state-based net neutrality statue that applies to broadband internet access services provided to customers in the state. The Ninth Circuit upheld the law as against a federal preemption challenge. *See* ACA Connects v. Bonta. (Full disclosure—I filed an amicus brief on behalf of a group of Internet Law scholars arguing against preemption in that case).

^106^*See* Verizon v. FCC at 651–652, 655, 658–659.

^107^2015 Open Internet Order ¶¶ 493–527; *see* 47 U.S.C. § 160(a).

^108^*See* Narechania (2022). It is worth noting that some states have moved on their own to introduce broadband rate regulation schemes, though they are being challenged as preempted by the federal Communications Act. *See, e.g*., New York State Telecomms. Assn. v. James (holding New York broadband affordability law preempted). (Full disclosure—I filed an amicus brief on behalf of a group of Internet Law scholars arguing against preemption in that case and the still-pending-as-of-this-writing appeal to the Second Circuit).

^109^As noted earlier, *see supra* note 6, policy interventions can also help complete the transition to technological abundance. For example, government can fund basic and applied research in telecommunications technology. These interventions are not mutually exclusive with those described above, but given the current state of technology my focus lies on policy interventions to reduce the economic barriers to abundant communications.

^110^*See* Cooper (2021).

^111^Pub. L. No. 117–158, 135 Stat. 429 (2021).
